# Increased IgG4 expression within tertiary lymphoid structures of esophageal cancer and implications for prognosis

**DOI:** 10.3389/fimmu.2025.1654655

**Published:** 2025-09-18

**Authors:** Chanjuan Su, Shuqi Chen, Xuqing He, Changchun Ma, Wei Huang, Chunyan Zhu, Hui Wang, Jiang Gu

**Affiliations:** ^1^ Provincial Key Laboratory of Molecular Pathology and Personalized Medicine, Center of Collaborative and Creative Center, Department of Pathology and Pathophysiology, Shantou University Medical College, Shantou, Guangdong, China; ^2^ Department of Radiation Oncology, Affiliated Cancer Hospital, Shantou University Medical College, Shantou, Guangdong, China; ^3^ Department of Pathology, The First People’s Hospital of Yunnan Province, The Affiliated Hospital of Kunming University of Science and Technology, Kunming, Yunnan, China; ^4^ Jinxin Research Institute for Reproductive Medicine and Genetics, Xinan Hospital for Maternal and Child Health Care, Chengdu, China

**Keywords:** esophageal cancer, immunoglobulin G4, tertiary lymphoid structure, tumor microenvironment, prognosis

## Abstract

**Background:**

Tertiary lymphoid structures (TLS) have been observed in various tumors, including esophageal squamous cell carcinoma (ESCC), where they function as active centers of humoral immunity within the tumor microenvironment, facilitating the production of distinct immunoglobulin (Ig) subtypes with diverse effects. This study aimed to investigate the expression of TLS-associated Ig in ESCC and its prognostic significance.

**Methods:**

A retrospective analysis was conducted on 109 ESCC cases. TLS presence was evaluated using immunohistochemistry, and the expression of Ig-positive cells was assessed. Additionally, gene expression profiles related to TLS were analyzed with gene chip data from 274 ESCC cases obtained from the GEO database.

**Results:**

Among the 109 ESCC cases, 69 (63.3%) exhibited TLS, while 40 (36.7%) did not. Patients with TLS demonstrated significantly better prognoses compared to those without TLS (P < 0.001). ESCC tumor tissues with TLS exhibited higher infiltration of Ig-positive immune cells. Among the variables examined, the differential expression of IgG4 was the most pronounced (P = 0.04). Furthermore, ESCC cases exhibiting TLS with elevated IgG4 expression demonstrated a poorer prognosis compared to those with TLS and reduced IgG4 expression (P = 0.036). The presence of TLS in general and low IgG4 expression in TLS were associated with improved survivals in patients who underwent post-surgery chemotherapy (p < 0.001 and p = 0.001, respectively). Analysis of the GEO dataset indicated that predominant IgG4 expression within TLS was associated with a reduced number of activated CD8+ T cells and an increased presence of CD4+ T helper cells and dendritic cells. The expression of TLS in ESCC was linked to a more favorable prognosis, whereas TLS with increased IgG4 expression correlated with a less favorable prognosis.

**Conclusions:**

The differential expression of immunoglobulins within TLS can modulate its function within the tumor microenvironment. In addition to the number of TLSs themselves, the immune heterogeneity present within TLS maybe a critical factor in individualized assessment of tumor immunotherapy.

## 1 Introduction

Esophageal carcinoma (EC) represents a formidable challenge within the landscape of digestive tract malignancies, particularly in China, where its incidence and mortality rates remain notably elevated. According to the 2020 GLOBOCAN global cancer data, the age-standardized incidence and mortality rates of EC worldwide were recorded at 6.3 and 5.6 per 100,000, respectively. However, within China, these Figures surged to 13.8 and 12.7 per 100,000, surpassing those observed in nations such as the United States and the United Kingdom (1, 2). Notably, within the Chaoshan region of China, the burden of EC stands prominently high, signifying a pressing public health concern (3). Due to the diversity of genetic characteristics and eating habits, squamous carcinoma is the most prevalent pathological type among Chinese people, which is different from adenocarcinoma pathology type more often seen in western countries.

Tumor immunotherapy is a rapidly developing field in cancer treatment. In tumor immune microenvironment (TIME), the interaction between tumor cells and immune cells or molecular components forms a complex system that plays a crucial role in tumor development, prognosis, and treatment. Tertiary lymphoid structures (TLS) are ectopic lymphoid tissues formed in abnormal lymphatic tissues or organ areas and are commonly seen in inflammatory reactions associated with autoimmune diseases, organ transplantation, infections, tumors, and are also known as ectopic lymphoid tissues (ELTs) or tertiary lymphoid organs (TLOs) (4). TLS has been reported in various tumors including malignant melanoma, colon cancer, breast cancer, gastric cancer, ovarian cancer, lung cancer, pancreatic cancer, liver cancer, and esophageal cancer, making it an important structure in TIME (5–12). TLS consists of germinal centers with B cells, T cell zones, and high endothelial venules and contains various cell types, including activated B cells, activated T cells, various types of T helper (Th) cells, follicular dendritic cells (FDCs), mature dendritic cells (DCs), natural killer (NK) cells, plasma cells, and macrophages, resembling the lymph follicle structure of secondary lymphoid organs (SLOs) (13, 14).

TLS is rich in activated B cell and plasma cell infiltration and serves as an active center of humoral immunity in TIME. Immunoglobulins (Ig) represent essential immune molecules orchestrating humoral responses, exerting critical anti-tumor effects through diverse mechanisms (15). Structurally, Ig can be categorized into five distinct types based on variations in their heavy chains: IgA, IgM, IgD, IgE, and IgG. IgG, the most prevalent subtype, further subdivides into four distinct forms (IgG1, IgG2, IgG3, and IgG4) characterized by unique structural and functional attributes. IgG1, deriving from IgM class switching, constitutes the predominant subtype, accounting for 43-75% of total IgG, and plays a paramount role in anti-tumor immunity. In contrast, IgG4, the least abundant subtype (0.8-11.7%), assumes a regulatory role, contributing significantly to immune suppression, tolerance, and autoimmune modulation (16). Different IgG subtypes have distinct biological functions, present in various immune cell types within TLS and play different roles in the TIME. However, the exact role of IgG subtype in TLS has not been illuminated.

This study investigated the distribution characteristics of TLS and the expressions of some relevant Ig subtypes in 109 cases of ESCC in the Chaoshan region of Guangdong Province, China. We examined TLS with immunohistochemistry (IHC) and investigated the impact of TLS and different Ig subtype expression patterns on the prognosis of ESCC. Additionally, we analyzed the molecular pathways associated with different immune expression patterns within TLS employing the GEO database.

## 2 Materials and methods

### 2.1 Clinical cohort and specimens

One hundred and nine cases of esophageal squamous cell carcinoma (ESCC) diagnosed between 2013 and 2019 were obtained at Cancer Hospital of Shantou University Medical College. Formalin-fixed and paraffin-embedded (FFPE) tissue blocks or slides from tumor tissues were collected, along with corresponding clinical and survival information. Basic clinical information and inclusion criteria are detailed in the **Supplementary Materials**. Overall survival (OS) and progression-free survival (PFS) were calculated using the surgery date as the start date and death, tumor recurrence, or metastasis as endpoints. All clinical case data and tissue specimens involved in this study received approval from the Medical Ethics Committee of Shantou University Medical College.

### 2.2 Immunohistochemistry staining and scoring

Consecutive sections of paraffin-embedded tissue were utilized for Hematoxylin and Eosin (HE), IHC, and Stain–Decolorize–Stain (SDS) experiments as previously described (17). The detailed antibody information in the article is presented in the **Supplementary Materials Supplementary Table S1**. IHC staining results were analyzed to comprehensively determine the presence of TLS in the tissue, assess the maturity of each TLS, and record their distribution. Serial sections of tumor tissues from each ESCC case were subjected to IgA (ZSGB-BIO, ZF-0305), IgM (ZSGB-BIO, ZF-0307), IgG1 (Abcam, ab201485), and IgG4(Abcam, ab109493) staining. After scanning with a digital slide scanner, five 200X tumor interstitial fields (including TLS fields) were randomly selected. The number of Ig-positive cells in each field was counted, averaged and divided by the field area (1.23 mm^2^ per 200X field), and then the average density of Ig-positive cells in each tissue in each case was finally obtained for subsequent analysis. IgG4 IHC staining was performed on tumor tissues from the 69 cases with TLS, and the same counting method was employed to obtain the average density of IgG4-positive cells. The number of positive cells per standard area was counted with Qupath (QuPath-0.4.3). The optimal cutoff value for the average density of IgG4-positive cells for survival analysis was calculated using X-tile (Version 3.6.1; Yale University), resulting in the classification of the 69 TLS-positive ESCC cases into IgG4 high and IgG4 low groups. The cutoff value for IgG4 high and low expression groups was set as 28.3 positive cells/200X field. The presence of TLS was assessed using a combination of morphological characteristics and specific biomarkers, including CXCL13, CD20, CD21, and Ki67, as detailed in **Table 1**. Immunoglobulin staining (IgA, IgM, IgG1, IgG4) was conducted on a subset of 15 cases, comprising 10 cases with TLS and 5 cases without TLS. A GC score of 0 was indicative of the absence of TLS, while a GC score of 1 denoted the presence of TLS without a germinal center. Conversely, a GC score of 2 signified the presence of TLS with an associated germinal center.

**Table 1:** Comparison of TLS characteristics in different developmental states.

Type	E-TLS	P-TLS	S-TLS
CD20	+	+	+
CD21	–	+	+
Ki67	+, few ki67 positivity	++, scattered ki67 positivity	+++, concentrated Ki67 positivity in GC
Irregular or small elliptical in shape	+	–	–
Follicular mesh-like structure	–	+	+
Germinal center	–	–	+

### 2.3 Immunoscore and statistical analysis

Immunoscores were calculated based on TLS maturity, germinal center (GC) presence, and IgG4 expression (18). Based on the staining results, we determined the presence of TLS and assigned a TLS score according to the following criteria: 0 (no TLS), 1 (Early TLS, E-TLS), 2 (Primary TLS, P-TLS), and 3 (Secondary TLS, S-TLS). Specifically, E-TLS was irregular or small elliptical in shape and exhibited positive CD20 aggregation. P-TLS was elliptical or ovoid in shape, positive for CD20 aggregation, CD21 positive, and formed a follicular mesh-like structure, with scattered ki67 positivity. S-TLS was elliptical or ovoid in shape, distinguishable into the LZ and DZ of the GC, positive for CD20 aggregation, CD21 positive, and formed a follicular mesh-like structure, with ki67 positivity concentrated in the GC. TLS-IgG4 score was defined as follows: 0 (no TLS), 1 (TLS with IgG4 low expression), and 2 (TLS with IgG4 high expression). The division into high and low IgG4 expression groups was based on the X-Tile results, as described previously (19). GC score was defined as follows: 0 (no GC), 1 (Containing more than one GC). The criteria for identifying GC was based on ki67 IHC results and morphological features. Statistical analyses were conducted using Prism (Version 9.0.0; GraphPad Software LLC), SPSS (Version 26.0.0.0; International Business Machines Corporation), and RStudio (Version 2022.02.3 + 492; RStudio, Inc.). Significance was set at α = 0.05 (P < 0.05).

### 2.4 Bioinformatic analysis

Gene chip sequencing data and clinical information for 274 ESCC biopsy tissues were downloaded from the GEO database (GEO, http://www.ncbi.nlm.nih.gov/geo). Single-sample Gene Set Enrichment Analysis (ssGSEA) was used to calculate gene expression signal scores for TLS-related gene sets, activated B cell-related gene sets, and immature B cell-related gene sets. The TLS-related gene set includes 12 chemokine signaling genes: CCL2, CCL3, CCL4, CCL5, CCL8, CCL18, CCL19, CCL21, CXCL9, CXCL10, CXCL11, CXCL13. The genes associated with activated B cells include ADAM28, CD180, CD79B, BLK, CD19, MS4A1, TNFRSF17, IGHM, GNG7, MICAL3, SPIB, HLA-DOB, IGKC, PNOC, FCRL2, BACH2, CR2, TCL1A, AKNA, ARHGAP25, CCL21, CD27, CD38, CLEC17A, CLEC9A, CLECL1. The gene set associated with immature B cells includes CD22, CYBB, FAM129C, FCRL1, FCRL3, FCRL5, FCRLA, HDAC9, HLA-DQA1, HVCN1, KIAA0226, NCF1, NCF1B, P2RY10, SP100, TXNIP, STAP1, TAGAP, ZCCHC24, and ZCCHC41. K-means clustering was performed on the 274 ESCC cases based on gene expression signal scores for the three gene sets mentioned. According to the clustering results, 274 ESCCs were divided into ESCC with low TLS gene expression signal (No TLS ESCC group) and high TLS gene expression signal (TLS ESCC group).

IGHG1 and IGHG4 gene expression levels were used to represent Ig subtypes IgG1 and IgG4 levels, respectively. Based on a 75% cutoff point in the gene expression levels of IGHG1 and IGHG4, the TLS ESCC group was further classified into four subgroups: a group with low expression of both IgG1 and IgG4 (1 IgG1 low/IgG4 low group), a group with low IgG1 expression and high IgG4 expression (2 IgG1 low/IgG4 high group), a group with high IgG1 expression and low IgG4 expression (3 IgG1 high/IgG4 low group), and a group with high expression of both IgG1 and IgG4 (4 IgG1 high/IgG4 high group).

The 28 immune cell gene sets were downloaded from the Tumor-immune system interactions database (TISIDB) (20, 21). The ssGSEA method was employed to calculate the signal scores for 28 immune cell gene sets in each case within the TLS group. Subsequently, differential statistical analyses were conducted for the 28 immune cell gene sets scores within the four specified groups above. Differential gene expression (DEG), Gene Ontology (GO) clustering analysis and Kyoto Encyclopedia of Genes and Genomes (KEGG) clustering analysis was conducted between IgG1 high/IgG4 low group and IgG1 low/IgG4 high group. Volcano plots and heatmaps for DEGs were generated, with the volcano plot created using the OmicStudio online plotting tool (https://www.omicstudio.cn/tool) and the heatmap generated using the SangerBox online plotting tool (http://www.sangerbox.com).

## 3 Results

### 3.1 Clinical characteristics and TLS distribution in ESCC specimens

Among the 109 cases, there were 89 males (81.7%) and 20 females (18.3%). The age range was 42 to 77 years, with a median age of 60 years and an average age of 59.9 years (95% CI: 58.5-61.3 years) (**Table 2**). Among the ESCC cases, there were 43 survivors (39.4%) and 66 deceased cases (60.6%). The mean survival time was 45.6 months (95% CI: 38.7-52.6 months), the median survival time was 40.0 months (95% CI: 27.0-53.0 months), the mean follow-up time was 58.2 months (55.5-60.9 months), and the median follow-up time was 56.0 months (51.6-60.4 months). Chi-square tests were conducted to analyze the correlation between the presence of TLS and gender, age, smoking history, alcohol consumption history, family history, tumor location, depth of infiltration, T stage, N stage, G stage, and pathological (pTNM) stage (**Table 2**). As shown in the table, categorical variables such as alcohol history (χ² = 9.923, P = 0.002), tumor location (χ² = 8.812, P = 0.041), T stage (χ² = 8.786, P = 0.029) were found to be correlated, while other categorical variables were independent of the presence of TLS.

**Table 2:** The clinical characteristics and TLS distribution table of 109 ESCC.

Clinical characteristics	ALL (N = 109)	No TLS group (N = 40)	TLS group (N = 69)	χ²	P value
Gender				1.443	0.230
Male	89(81.7%)	35(87.5%)	54(78.3%)		
Female	20(18.3%)	5(12.5%)	15(21.7%)		
Age at surgery (years)				0.687	0.407
Median	60	61	59		
Mean	59.9	59.8	59.9		
Range	42-77	42-76	44-77		
<60	52(47.7%)	17(42.5%)	35(50.7%)		
≥60	57(52.3%)	23(57.5%)	34(49.3%)		
Tabacco use				1.071	0.301
No	28(25.7%)	8(20.0%)	20(29.0%)		
Yes	81(74.3%)	32(80.0%)	49(71.0%)		
Alcohol use				9.923	0.002*
No	57(52.3%)	13(32.5%)	44(63.8%)		
Yes	52(47.7%)	27(67.5%)	25(36.2%)		
Genetic disorders				0.460	0.498
No	92(84.4%)	35(87.5%)	57(82.6%)		
Yes	17(15.6%)	5(12.5%)	12(17.4%)		
Tumor location^**^				8.812	0.041*
Cervical	1(0.9%)	0(0.0%)	1(1.4%)		
U	12(11.0%)	2(5.0%)	10(14.5%)		
M	66(60.6%)	22(55.0%)	44(63.8%)		
L	25(22.9%)	15(37.5%)	10(14.5%)		
Multiple	5(4.6%)	1(2.5%)	4(5.8%)		
Depth of infiltration^**^				6.960	0.056
Mucosal layer	3(2.8%)	0(0.0%)	3(4.3%)		
Submucosal layer	8(7.3%)	0(0.0%)	8(11.6%)		
Muscularis propria	10(9.2%)	4(10.0%)	6(8.7%)		
Adventitia	88(80.7%)	36(90.0%)	52(75.4%)		
The 8th edition AJCC T staging^**^				8.786	0.029*
T = 1	11(10.1%)	0(0.0%)	11(15.9%)		
T = 2	10(9.2%)	4(10.0%)	6(8.7%)		
T = 3	42(38.5%)	19(47.5%)	23(33.3%)		
T = 4	46(42.2%)	17(42.5%)	29(42.0%)		
The 8th edition AJCC N staging				3.174	0.366
N = 0	35(32.1%)	12(30.0%)	23(33.3%)		
N = 1	38(34.9%)	12(30.0%)	26(37.7%)		
N = 2	20(18.3%)	7(17.5%)	13(18.8%)		
N = 3	16(14.7%)	9(22.5%)	7(10.2%)		
The 8th edition AJCC G staging^**^				0.437	0.852
G = 1	45(41.3%)	15(37.5%)	30(43.5%)		
G = 2	51(46.8%)	20(50.0%)	31(44.9%)		
G = 3	13(11.9%)	5(12.5%)	8(11.6%)		
Pathological stage (pTNM stage)^**^				6.774	0.081
I	9(8.3%)	0(0.0%)	9(13.0%)		
II	21(19.3%)	7(17.5%)	14(20.3%)		
III	49(45.0%)	21(52.5%)	28(40.6%)		
IV	30(27.5%)	12(30.0%)	18(26.1%)		
Survival status				10.007	0.002*
0	43(39.4%)	8(20.0%)	35(50.7%)		
1	66(60.6%)	32(80.0%)	34(49.3%)		

*The significance level was α = 0.05 and P < 0.05 was statistically significant.

**The minimum expected frequency is 5. The Monte Carlo simulation method is used to test, and the simulation sample number is 100000.

### 3.2 The characteristics of TLS in ESCC

**Figure 1:**
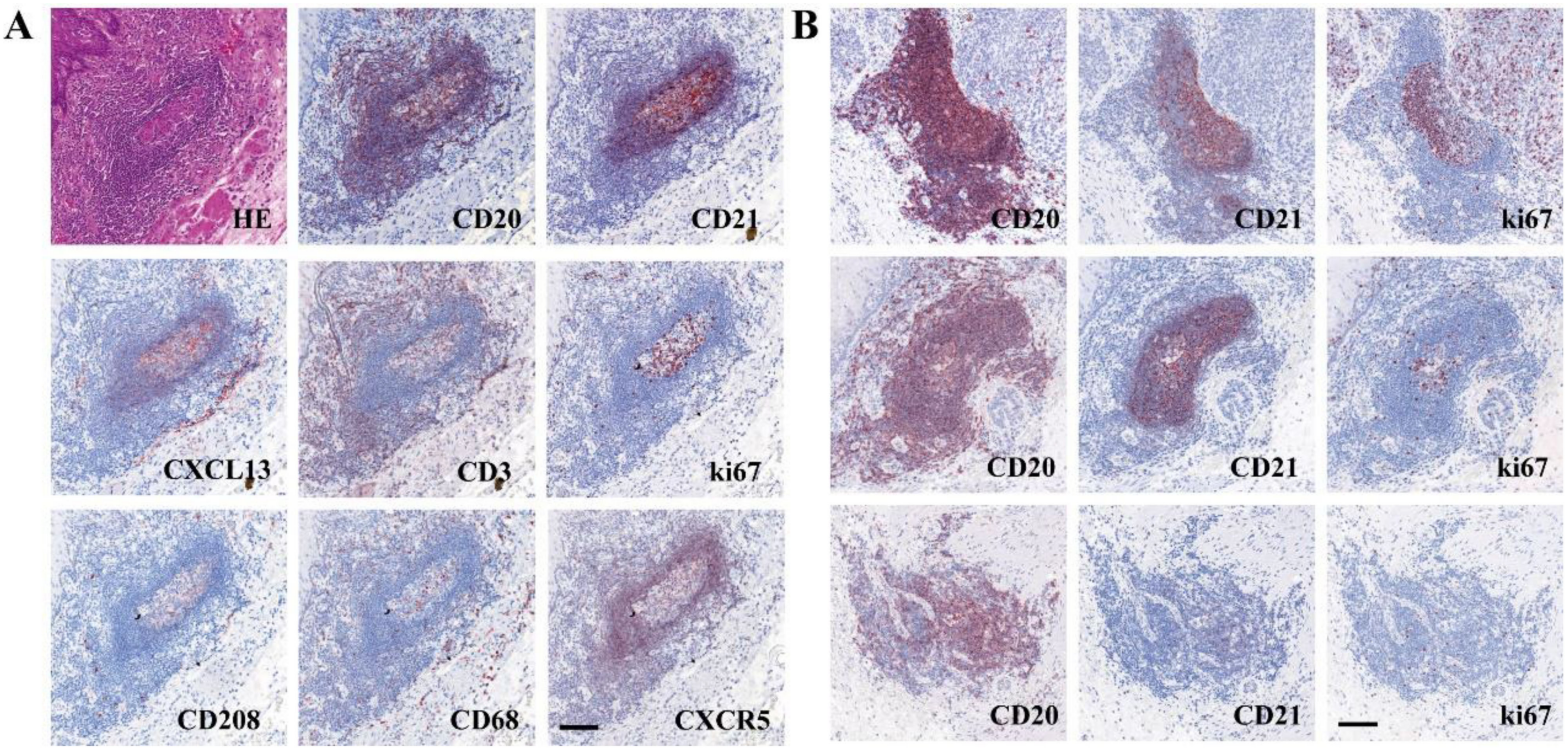
The characteristic of TLS in ESCC and tumor margin. **(A)** Composition of a typical TLS in tumor margin (The sequence of SDS in section 1 was CD20-CD21-CXCL13-CD3, and that in section 2 was ki67-CD208-CD68-CXCR5). **(B)** Different levels of maturity of TLS (top, S-TLS; middle, P-TLS; bottom, E-TLS). Scale bar=100μm.

Microscopic observation of the distribution of various immune cells within TLS (**Figure 1A**) revealed mature TLS structures resembling secondary lymphoid follicles in lymph nodes, with distinguishable T cell zones and B cell zones, including the light zone (LZ) and the dark zone (DZ) in GC. B cell zones exhibited abundant CD20^+^ B cells, CD21^+^ FDCs, and positive signals for CXCL13 and CXCR5 chemotactic factors. The peripheral T cell zones surrounding the B cell zones were infiltrated by numerous T cells and Th cells. Mature DCs were distributed in the periphery of the T cell zones. Macrophages were distributed both within the GC and in the periphery of the T cell zones. Based on the IHC results for CD20, CD21, and ki67, TLS were classified into three distinct maturation stages (**Figure 1B**): ① Early TLS (E-TLS); ② Primary TLS (P-TLS); and ③ Secondary TLS (S-TLS). E-TLS were irregular or small elliptical in shape and exhibited positive CD20 aggregation. P-TLS were elliptical or ovoid in shape, positive for CD20 aggregation, CD21 positive, and formed a follicular mesh-like structure, with scattered ki67 positivity. S-TLS were elliptical or ovoid in shape, distinguishable into the LZ and DZ of the GC, positive for CD20 aggregation, CD21 positive, and formed a follicular mesh-like structure, with ki67 positivity concentrated in the GC. And the indicators and structural characteristics of matures S-TLS were similar to the lymph node structure of tumor patients (**Supplementary Figure S1**). The comparison of cellular markers and morphological characteristics of TLS shown as **Table 1**.

**Figure 2:**
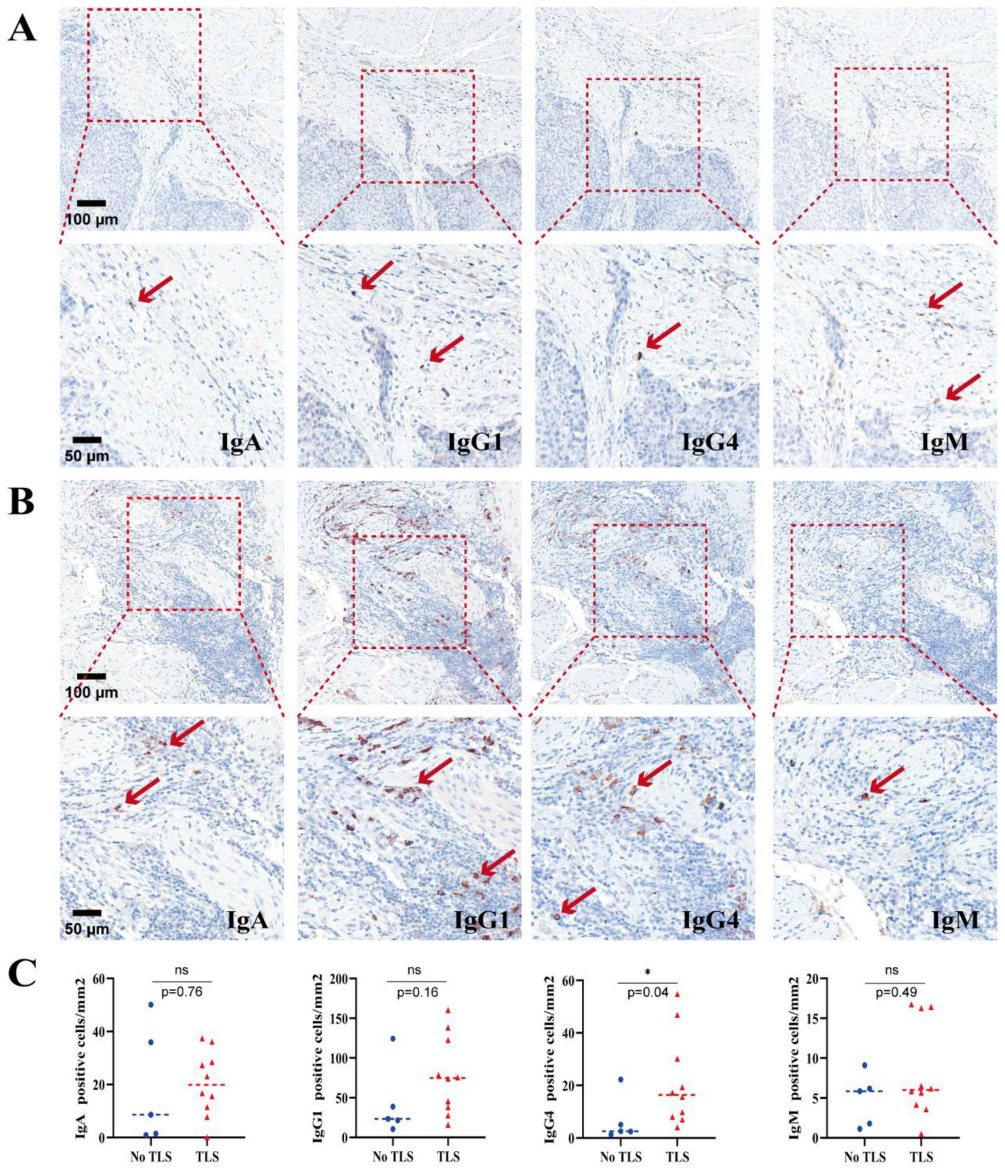
**(A)** The expression of each Ig subtype of No TLS-ESCC. **(B)** The expression of each Ig subtype of TLS-ESCC. **(C)** Comparison of Ig subtype expression in tumor tissues with and without TLS in ESCC (n=15). NS represents no statistical significance, *P < 0.05.

IgA, IgM, IgG1, and IgG4 stainings were analyzed on tumor tissues from 15 ESCC cases. Among these, 10 cases had TLS, while 5 cases did not (**Figures 2A, B**). Non-parametric t-tests (Mann-Whitney U tests) were used to compare the average densities of different Ig-positive cells between T tissues with TLS and those without TLS. The results showed that tumor tissues with TLS had higher infiltration of IgG-positive cells compared to tumor tissues without TLS (**Figure 2C**). Among them, the median of IgG4-positive cells showed the most significant difference, with statistical significance (No TLS group = 2.602/mm², TLS group = 16.42/mm², P = 0.0400); while the median of IgA, IgM, and IgG1-positive cells showed higher infiltration but without statistical significance (IgA: No TLS group = 8.618/mm², TLS group = 19.92/mm², P = 0.7679; IgM: No TLS group = 5.845/mm², TLS group = 6.016/mm², P = 0.4968; IgG1: No TLS group = 23.58/mm², TLS group = 74.80/mm², P = 0.1645) (**Figure 2C**).

### 3.3 The prognosis of TLS in ESCC clinical cohort

**Figure 3:**
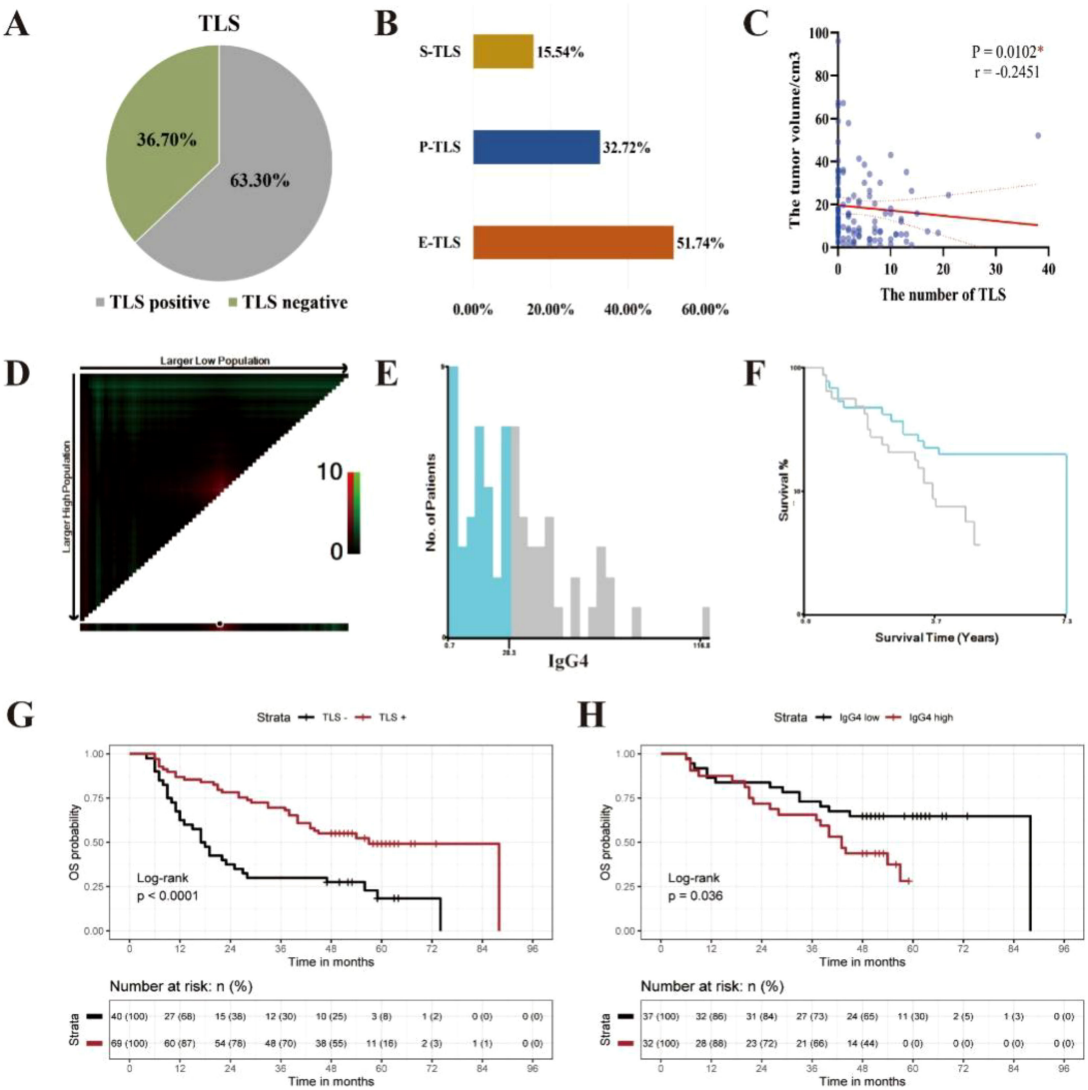
The clinical prognosis of TLS in ESCC clinical cohort. **(A)** The distribution of TLS in ESCC. **(B)** The maturity of TLS in ESCC. **(C)** The correlation between the quantity of TLS and tumor volume in ESCC. **(C)** The correlation between the quantity of TLS and tumor volume in ESCC( *P < 0.05). **(D)** Distribution of χ2 values for the average positive density of IgG4 containing B cells. **(E)** Histogram displaying the distribution of IgG4 average positive density. **(F)** KM survival curve for the optimal cutoff value of IgG4 average positive density, with a cut-off value of 28.3/mm^2^. **(G)** Kaplan-Meier survival curves between TLS negative and TLS positive group in 109 cases of ESCC (***p<0.0001). **(H)** Kaplan-Meier survival curves between IgG4 low and IgG4 high group in 69 cases of ESCC with TLS (*p<0.05).

Staining results for CXCL13, CD20, CD21, and ki67, the positivity rate, distribution quantity, distribution location, and maturity of TLS were statistically analyzed in 109 ESCC cases. Among them 69 cases (63.3%) were found to have TLS, while 40 cases (36.7%) did not (**Figure 3A**). There were a total of 489 TLS in the 109 ESCC cases, including 253 E-TLS (51.8%), 160 P-TLS (32.7%), and 76 S-TLS (15.5%) (**Figure 3B**). The average number of TLS per case was 4.45 (95% CI: 3.36-5.61), with a median of 2 TLS and a range from 0 to 38 TLS. The immunoscore for TLS: TLS score = 0, was observed in 40 cases (36.70%), TLS score = 1 in 18 cases (16.51%), TLS score = 2 in 21 cases (19.27%), and TLS score = 3 in 30 cases (27.52%). The immunoscore for GC presence: GC score = 0, was found in 40 cases (36.70%), GC score = 1 in 39 cases (35.78%), and GC score = 2 in 30 cases (27.52%).

Spearman correlation analysis was used to analyze the correlation between the number of TLS and tumor volume size. Tumor volume size was defined as the product of the length, width, and thickness of the tumor, as recorded in the clinical pathology report (mm³). The results showed that the number of TLS in ESCC was significantly negatively correlated with tumor volume size (**Figure 3C**) and was statistically significant at a significance level of α = 0.05 (P = 0.0102, r = -0.2451, 95% CI: -0.4188, -0.05413).

The log-rank test was used to assess the significance of differences in survival time among different clinical indicator groups and the presence or absence of TLS in the 109 ESCC cases. The results showed that N stage (P < 0.001), pTNM stage (P = 0.001), alcohol history (P = 0.023), and the presence of TLS (P < 0.001) had significant differences in terms of OS (**Figure 3G**, **Supplementary Figure S2**).

The optimal cut off value for the average density of IgG4-positive cells was calculated with X-tile software to be 28.3/mm^2^. Among the 69 ESCC cases with TLS, they were divided into IgG4 high and IgG4 low groups, with 32 cases in the IgG4 high group (46.4%) and 37 in the IgG4 low group (53.6%) (**Figures 3D–F**). The log-rank test was used to assess the differences in survival time among different groups based on TLS score, GC score, and IgG4 group in the 69 ESCC cases with TLS. The results showed that the IgG4 group (P = 0.036) had statistically significant differences in OS, while TLS score (P = 0.698) and GC score (P = 0.442) did not show statistically significant difference in OS (**Figure 3H**, **Supplementary Figure S3**). Subsequently, we performed univariate Cox survival analyses on multiple clinicopathological parameters. The results demonstrated that a higher TLS score was associated with improved patient prognosis. Similarly, the GC score also exhibited a favorable prognostic indication. Interestingly, in the TLS-IgG4 scoring system, both the TLS-Low-IgG4 and TLS-High-IgG4 groups showed better prognoses compared to the non-TLS group, suggesting that the presence of TLS itself may exert a greater influence on prognosis than IgG4 expression levels alone (**Supplementary Table S2**). Furthermore, when comparing these three scoring systems with the classical pathological prognostic indicator pTNM, both the TLS score and GC score remained positively associated with favorable patient outcomes in multivariate Cox survival analyses. However, within the TLS-IgG4 scoring system, only the TLS-Low-IgG4 group demonstrated a statistically significant association with favorable prognosis, further supporting the notion that high IgG4 expression may, to some extent, attenuate the beneficial prognostic effect of TLS (**Supplementary Table S3**).

### 3.4 The impact of TLS and IgG4 expression on prognose in different postoperative treatment patients

**Figure 4:**
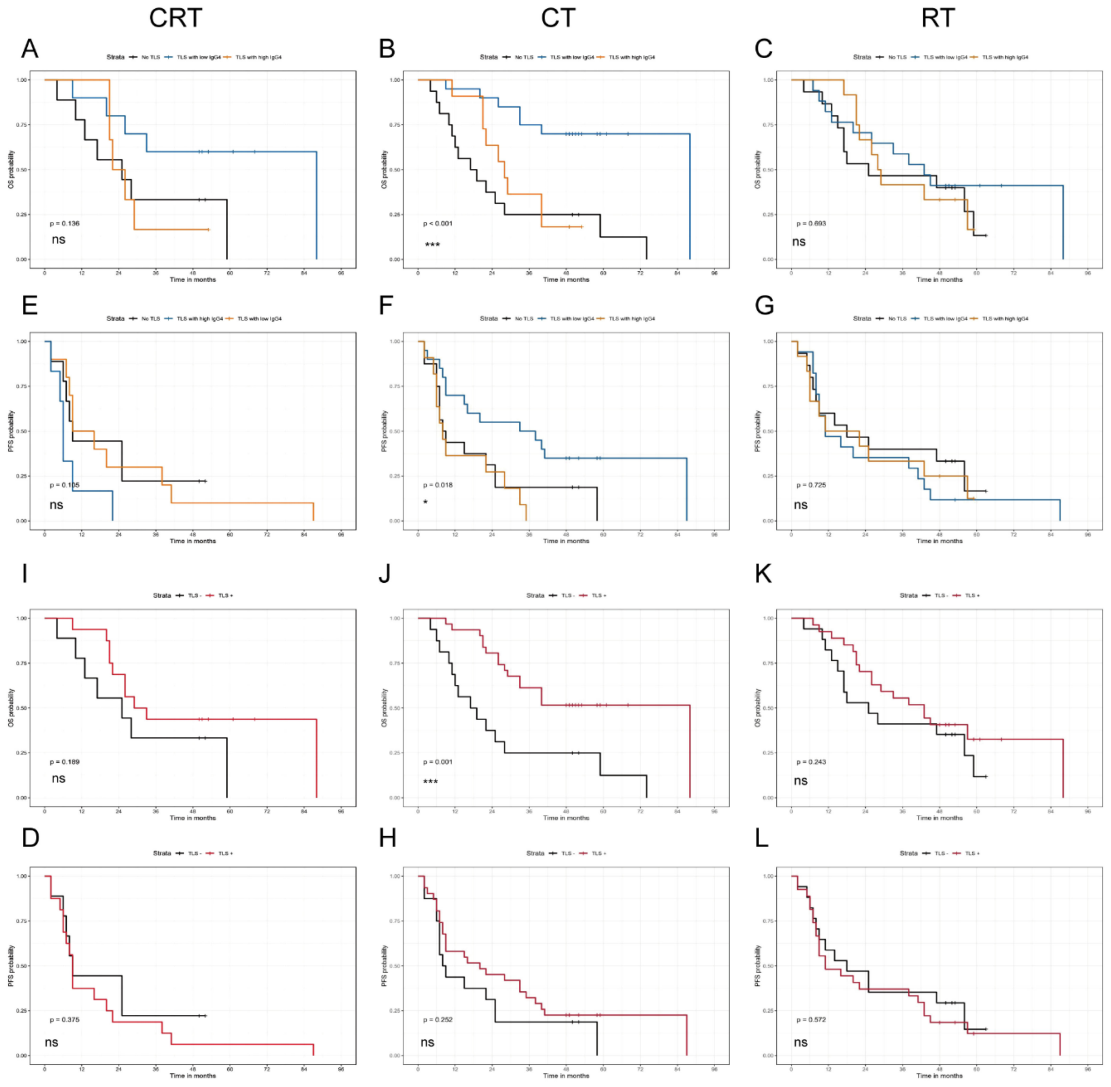
The influence of TLS and IgG4 on prognosis including OS and PFS statistics, in different postoperative treatment groups: chemotherapy combined with radiotherapy group (CRT, **A, E, I, D**), chemotherapy group (CT, **B, F, J, H**), radiotherapy group (RT, **C, G, K, L**). (*P < 0.05, and **P <0.001).

Out of the 109 patients, 1.8% (2/109) received immunotherapy combined with chemotherapy, and 1.8% (2/109) received immunotherapy combined with radiotherapy and chemotherapy. Immunotherapy drugs included Camrelizumab, with treatment regimens comprising Camrelizumab in conjunction with Tegafur, Gimeracil and Oteracil Potassium Capsules or Camrelizumab combined with paclitaxel and carboplatin. These four patients remained alive and were followed up until July 2024. Among 109 patients, 21% (23/109) received combined radiotherapy and chemotherapy, 17% (19/109) underwent post-operative radiotherapy, 18% (20/109) received postoperative chemotherapy, and the remaining 39% (43/109) did not receive any corresponding treatment after surgery. The impact of TLS and IgG4 on prognosis across different postoperative treatment groups is illustrated in **Figures 4A–L**. Notably, patients in the high TLS level group exhibited a more favorable prognosis following chemotherapy compared to those in the no TLS group (p=0.001, **Figure 4J**). Furthermore, within the chemotherapy group, the survival prognosis of patients in the low-level IgG4 TLS group was superior to that of both the high-level IgG4 TLS group and the no TLS group (p<0.001, **Figure 4B**). And the number of patients receiving immunotherapy in this queue is relatively small, and therefore the group differences were not treated statistically.

### 3.5 The gene expression profile of TLS in ESCC GEO database

**Figure 5:**
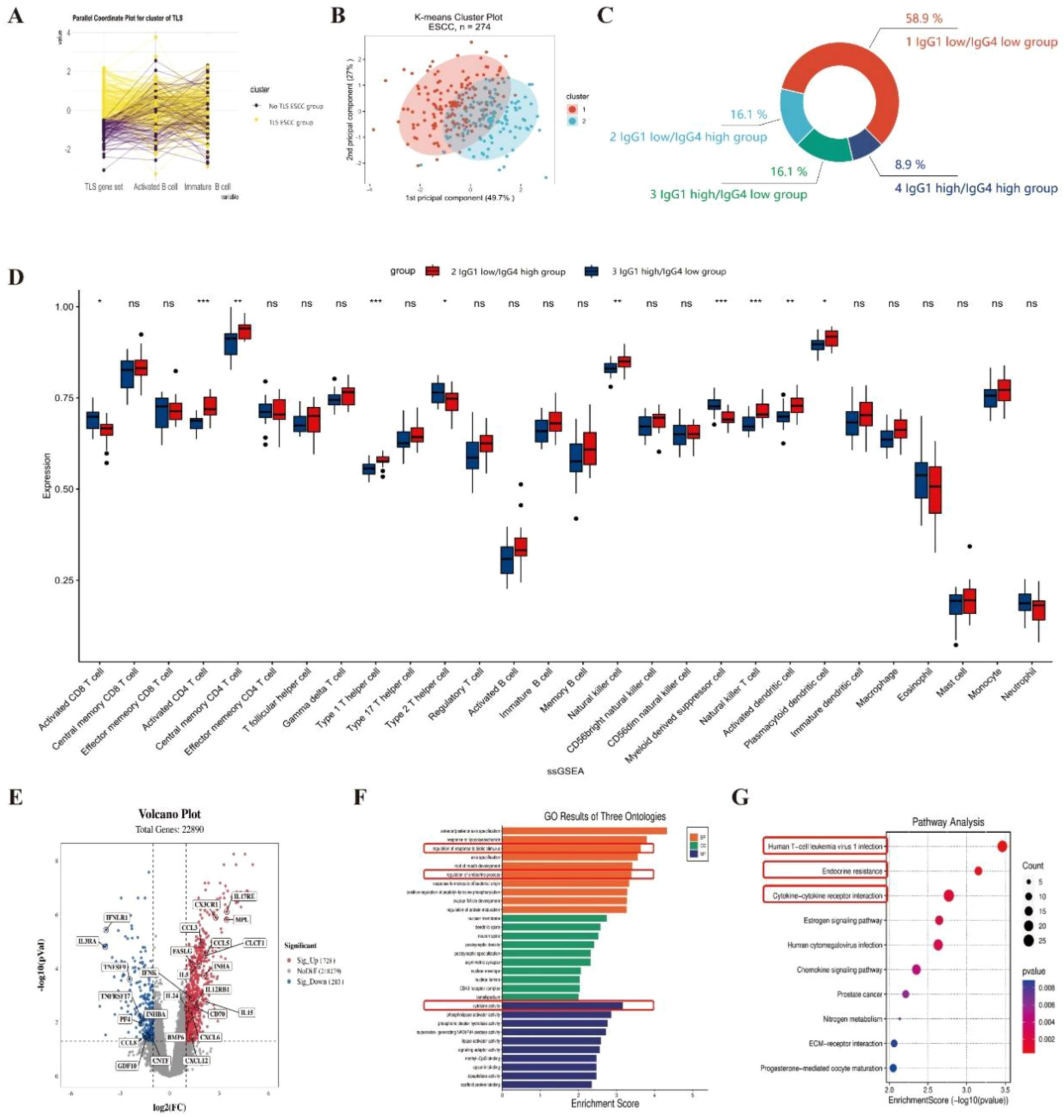
The gene expression profile of TLS in ESCC GEO database cohort. **(A)** ssGSEA gene set scores. **(B)** K-means clustering diagram based on mean values. **(C)** The distribution of four subgroups in the TLS ESCC group. **(D)** The differential expression of 28 immune cells between “2 IgG1 low/IgG4 high group” and “3 IgG1 high/IgG4 low group”. **(E)** The volcano plot comparing the differential expression of genes between “2 IgG1 low/IgG4 high group” and “3 IgG1 high/IgG4 low group”. **(F)** The GO enrichment pathways of the differentially expressed genes. **(G)** The KEGG enrichment pathways of the differentially expressed genes.

Sequencing data files and gene annotation files for the “GSE69925” dataset were downloaded from the GEO database, resulting in a gene expression matrix of 22,892 genes for 274 ESCC cases after data cleaning. The gene expression signal scores for TLS-related gene sets, activated B cell-related gene sets, and immature B cell-related gene sets were calculated using the ssGSEA method. Based on the gene expression signal scores from these three gene sets, K-means clustering was performed on the 274 ESCC cases (**Figure 5A**). The Hopkins statistic was 0.709, indicating clustering significance. The optimal number of clusters was determined to be 2 using the Silhouette coefficient method (**Supplementary Figure S4**). Among the 274 ESCC cases, 124 cases (45.3%) were divided into TLS ESCC group with high expression of TLS gene sets, and 150 cases (54.4%) were divided into No TLS ESCC group with low TLS gene expression signal (**Figure 5B**). By grouping the expression of IgG1/IgG4, it was found that 58.9% of the samples had both low expressions of IgG1 and IgG4 (**Figure 5C**).

The gene expression signal scores for 28 immune cell-related gene sets were calculated using ssGSEA for the TLS ESCC group. ANOVA and LSD tests were used to compare the differences in immune cell infiltration among four subgroups in the TLS ESCC group (**Figure 5D**, **Supplementary Figure S5–S10**). The “2 IgG1 low/IgG4 high group” had fewer infiltrating activated CD8^+^ T cells (P = 0.008), type 2 T helper cells (P = 0.040), and myeloid-derived suppressor cells (P < 0.001), but more infiltrating activated CD4^+^ T cells (P < 0.001), central memory CD4^+^ T cells (P = 0.004), type 1 T helper cells (P < 0.001), natural killer cells (P = 0.033), natural killer T cells (P < 0.001), activated DCs (P = 0.001), and plasma-like DCs (P = 0.042) (**Figure 5D**). The “2 IgG1 low/IgG4 high group” also had more central memory CD4^+^ T cells (P = 0.007), type 1 T helper cells (P = 0.050), regulatory T cells (P = 0.049), activated B cells (P < 0.001), natural killer T cells (P < 0.001), plasma-like DCs (P = 0.006), macrophages (P = 0.032), and neutrophils (P = 0.004) compared to the “1 IgG1 low/IgG4 low group” (**Supplementary Figure S6**). These results suggested that ESCC with TLS and predominant IgG4 expression had reduced tumor killing capabilities.

Using differential gene expression (DEG) analysis, genes that showed significant differences were screened using “2 IgG1 low/IgG4 high group” as the experimental group and “3 IgG1 high/IgG4 low group” as the control group, excluding IGHG1 and IGHG4 genes. A total of 1,011 DEGs were obtained, including 728 upregulated genes and 283 downregulated genes (**Figure 5E**). GO clustering analysis and KEGG clustering analysis were used to analyze the enriched pathways among the 1,011 DEGs (**Figures 5F, G**). According to the combined GO and KEGG clustering results, the differentially expressed genes in “2 IgG1 low/IgG4 high group” were primarily enriched in pathways related to the regulation of response to biotic stimulus (GO pathway: regulation of response to biotic stimulus; KEGG: Human T-cell leukemia virus 1 infection), endocrine resistance (GO: regulation of endocrine process; KEGG: Endocrine resistance), and cytokine activity (GO: cytokine activity; KEGG: Cytokine-cytokine receptor interaction).

## 4 Discussion

The advancements of tumor immunotherapy have been increasingly applied in clinical practice. The importance of TIME in the occurrence, development, and prognosis of tumor and response to treatment has been increasingly appreciated. TLS is an ectopic lymphoid structure formed in the inflammatory response of autoimmune diseases, organ transplantation, infection, tumors, and other diseases. Recently, the distribution characteristics of TLS in different types of tumors, its impact on tumors, its relationship with clinical prognosis and treatment efficacy, and its application as a potential target in tumor immunotherapy have been investigated by a few groups (22–25).

Most studies suggested that TLS is associated with a good prognosis and can enhance the efficacy of immune therapy. Zhou, et al, found that bladder cancer with high expression of TLS-related genes in the gene expression profiles of 11,835 bladder cancer cases from the TCGA database had favorable prognosis and better responses to PD-1 immune checkpoint inhibitors (25). Vanhersecke et al, found that the presence of mature TLS improved the objective response rate, prolonged progression-free survival, and TLS was independent of PD-L1 expression level and the amount of infiltrating CD8^+^ T cells in primary pancreatic cancer patients who received anti-PD-1/PD-L1 antibody therapy (24). These studies suggest that TLS played an important role in anti-tumor immunity and can influence the efficacy of immunotherapy. However, there are also reports suggesting that TLS might be associated with tumor recurrence, metastasis, and immune escape. Siyuan Dai et al. reported that the infiltration of TLS-associated CXCL13^+^CD8^+^ T cells in the tumor stroma may lead to an immune-suppressive microenvironment, which promoted tumor immune evasion and progression, resulting in poor prognosis (26). In another relatively large group of esophageal cancer, the number of mature TLS is believed to be positively correlated with better prognosis (27). In this study, clinical information, prognosis, and paraffin-embedded tissue specimens of ESCC resected from patients at the Cancer Hospital of Shantou University Medical College from 2013 to 2019 were retrospectively examined. The relationship between TLS and prognosis of ESCC was investigated with IHC. We found that survival prognosis is correlated with TLS and ESCC, where TLS tends to have a better survival prognosis (69 ESCC with TLS vs 40 ESCC without TLS: OS, P < 0.001, PFS, P = 0.018). However, our study did not find that a higher degree of maturity in TLS results in a better survival prognosis (TLS score: OS, P = 0.698, PFS, P = 0.832; GC score: OS, P = 0.442, PFS, P = 0.289). In cases where the IgG4 subtype of TLS-related Ig is highly expressed, TLS does not lead to a better prognosis (32 ESCC with TLS and IgG4 expression high vs 37 ESCC with TLS and IgG4 expression low: OS, P = 0.036). In our study, morphological maturity of TLS was not associated with patient prognosis, but the IgG4-subclass differentiation of B cells in TLS was associated with poor prognosis. This also suggests that TLS has its own polarization direction and functional bias.

The heterogeneity of TLS has been consistently observed across multiple studies. TLS differing in maturity, density, and spatial location may exert opposing effects on antitumor immunity. TLS are thought to enhance responses to immunotherapy. For example, TLS improved survival in patients receiving immune checkpoint inhibitors by promoting antibody production and T cell activation in renal cell carcinoma (28). Similarly, TLS may serve as reliable predictive biomarkers for immunotherapy efficacy in solid tumors (28, 29). Furthermore, TLS density was significantly associated with immune responses and memory T cell formation following neoadjuvant immunotherapy (30). TLS maturation can be modulated by regulating tryptophan metabolism, and interventions such as tryptophan-restricted diets or TDO2 inhibitors have been shown to increase mature TLS formation and synergize with anti–PD-1 therapy. These findings provide insights for developing novel immunotherapeutic strategies in HCC and other malignancies (31). The heterogeneity of TLS—encompassing its density, maturity, and spatial organization—collectively shapes its antitumor immune function. This structural and functional variability may offer guidance to personalized immunotherapy strategies (13, 32–34).

Our findings demonstrate that TLS exerts a substantial impact on patient survival, and that specific features such as B-cell class switching to IgG4 further modulate prognosis. These observations underscore the functional heterogeneity of TLS and the intricate nature of the immune microenvironment. Perhaps this can also explain why TLS’s prognostic situations were not all alike in recent studies (4). There have been reports that different cell subtypes within TLS may have different immune functions. In a study by Yamaguchi et al, TLS was classified into five dominant cell type groups (GC-TLS type, B cell-rich type, FDC-rich type, Th-rich type, and CTL/B/Th type) based on the proportions of these six immune cell types (cytotoxic T cells, GC B cells, Th cells, B cells, FDCs, and macrophages). The TLS dominated by Th cells was associated with tumor recurrence (35). However, there is limited research on the expression characteristics of TLS-related Ig subtypes. Chudakov et al, found in an animal experiment that TLS is the main site for the class switch of IgE, rather than in secondary lymphoid organs (15). Meylan et al, studied the response of B cells within TLS in renal cell carcinoma through spatial transcriptomics and found that IgG- and IgA-secreting plasma cells could move toward the tumor site along fibroblasts; and tumors positive for TLS had higher activity of IgG-secreting plasma cells and anti-tumor activity (28). In this study, IHC was performed on tumor tissue, pericarcinomatous tissue, and lymph node tissue of 15 cases of ESCC to stain for IgA, IgM, IgG1, IgG4, and the average positive density for each was calculated. The aim was to compare the differences in average density of Ig-positive cells in the tumor tissue of ESCC with and without TLS, and to investigate the impact of TLS on Ig infiltration in ESCC. Based on our previously published research (36–38), IgG4 is significantly elevated in esophageal cancer patients and closely related to immune suppression, which is the primary focus of this study, and the levels of IgG2 and IgG3 are comparatively low, and their antitumor roles are relatively minor or unknown (39). Therefore, we only included IgG1 (account for 50% in four IgG subclasses), which played a significant role in tumor immunity, and compared it with IgG4 in this study. The results showed that ESCC tumor tissue with TLS had higher levels of Ig infiltration compared to tumor tissue without TLS, and tumor tissue without TLS had lower expression levels of all Ig subtypes. Among them, the expression difference of IgG4 was the most significant (the median of IgG4-positive cells: No TLS = 2.602/mm2, TLS = 16.42/mm2, P = 0.0400). Based on the above reports and results, the cell subtypes in TLS within tumors are complex, potentially exhibiting both anti-tumor and pro-tumor effects through certain mechanisms. Among these, the different subtypes of Ig secreted by activated B cells within TLS may have varying impacts on the prognosis of tumor survival, which may be attributed to the distinct immunological functions exerted by different IgG subtypes. Low-level IgG4 in TLS and TLS expression in chemotherapy patients after surgery indicates better prognosis suggests that immune infiltration has an important impact on the overall treatment of patients. In recent years, immunotherapy combined with chemotherapy has become the first-line treatment for refractory and recurrent esophageal cancer, and has achieved encouraging results in clinical practice (40). Chemotherapy can rapidly induce tumor antigens releasing. In the group of patients with high levels of immune infiltration, antigen stimulation promotes a strong immune response, thereby promoting better prognosis, suggesting that the presence of TLS is an enhancer for anti-tumor immunity.

The immunosuppressive role of IgG4 in tumors is not yet fully understood. However, recent investigations have provided important insights into its underlying mechanisms. As early as 2013, a study on malignant melanoma demonstrated that tumor-induced Th2-type inflammation promotes the accumulation of CD22+ B cells and IgG4+ infiltrating cells within tumors, leading to the polarization of tumor-associated B cells to produce IgG4 (41, 42). Unlike IgG1, tumor antigen-specific IgG4 is ineffective at triggering effector cell-mediated tumor killing and can inhibit IgG1-mediated antitumor effects by reducing FcγRI activation. In a human melanoma xenograft mouse model, IgG4 significantly reduced the tumoricidal efficacy of IgG1, and serum IgG4 levels correlated negatively with patient survival. Our recent research has further revealed concurrent increases in IgG4 and IL-10 levels, promoting the formation of an immunosuppressive microenvironment. At the cellular level, IgG4 can inhibit classical antitumor immune effects, including antibody-dependent cell-mediated cytotoxicity (ADCC) and antibody-dependent cellular phagocytosis (ADCP), which are mediated by antitumor IgG1. Moreover, our animal experiments confirmed that IgG4 reshapes the immune microenvironment by enhancing M2-type macrophage polarization, reducing CD8+ T-cell infiltration, and elevating the expression of various anti-inflammatory cytokines, collectively facilitating tumor growth (36–38). Subsequent investigations further uncovered that glutathione (GSH) can disrupt disulfide bonds in IgG4 heavy chains, enhancing the Fc-Fc interaction between IgG4 and immobilized IgG subtypes. The combined application of IgG4 and GSH markedly augmented their inhibitory effects on classical ADCC, ADCP, and complement-dependent cytotoxicity (CDC), thus leading to local immunosuppression and promoting tumor progression (38). Collectively, these findings indicate that IgG4 suppresses tumor immunity through multiple distinct pathways.

Furthermore, in this study, the GEO database was utilized to study and analyze the gene expression profile related to TLS in ESCC, to further explore the mechanism of the high expression of IgG4 in TLS and its impact on the TIME. The results indicate that high expression of TLS-related IgG4 is associated with less infiltration of activated CD8^+^ T cells, type 2 T helper cells, and more infiltration of activated CD4^+^ T cells, type 1 T helper cells, natural killer cells, and natural killer T cells. The reduced number of activated CD8^+^ T cells in TLS predominantly expressing IgG4 may result in decreased tumor-specific killing ability and thus lead to poor prognosis. We also preliminarily validated this viewpoint in another research article with multi-color fluorescence staining method on tumor tissues (43). The relationship between IgG4 and CD8+ T cells in the tumor microenvironment remains unclear and warrants further investigation. Our preliminary studies have shown that IgG4 can inhibit T cell activity and proliferation during *in vitro* T cell culture. In addition, through multiple fluorescence staining experiments, we observed a substantial expression of IL-10 within TLS, predominantly originating from IL-10-positive regulatory T and B cells (Treg/Breg). It was also found that IgG4 has the potential to promote macrophage differentiation towards M2 with *in vitro* culture and animal models (37, 43). M2 macrophages exert an inhibitory immunity through various pathways, including secretion of IL-10. It has also been reported that in the tumor microenvironment of breast cancer, B cells can promote the expression of IgG4 by up regulating the level of IL-10 (44). IL-10 can promote the formation of an immunosuppressive microenvironment and facilitate T cell immune suppression and tumor escape (45–48). Based on our current results and existing literature, we speculate that IgG4 can promote production of IL-10, thereby constituting an immune microenvironment that is more inclined towards TH2 type conversion, thereby inhibiting T cell expression and function. DEG differential gene analysis revealed 729 upregulated genes and 284 downregulated genes in the group with low expression of IgG1 and high expression of IgG4 compared to the group with high expression of IgG1 and low expression of IgG4. GO clustering analysis showed enrichment in pathways such as regulation of response to biotic stimulus, regulation of endocrine process, and cytokine activity. KEGG clustering analysis showed significant enrichment of differential genes in pathways such as Human T-cell leukemia virus 1 infection, Endocrine resistance, and Cytokine-cytokine receptor interaction.

Previous research reported increase of IgG4 levels in the context of chronic infections, immune tolerance, and autoimmune diseases (39). In our previous studies, we found that IgG4 was highly expressed in the serum of ESCC patients and was associated with poor survival. In prior studies, we conducted a comparative analysis of the relationship between IgG4 expression levels within the TLS field of view across multiple collected images and patient prognosis. In the present study, we performed an analysis of IgG4 expression in 69 TLS-positive cases and whole slice, employing a more comprehensive analytical strategy than previously utilized. Consequently, this approach yielded divergent trends (49). Gao et al, analyzed the infection status of Porphyromonas gingivalis (Pg) and the prognosis of 312 ESCC patients who received neoadjuvant chemotherapy. They found that Pg infection may lead to poor prognosis for ESCC, affect the efficacy of chemotherapy, and promote tumor invasion and progression (50). Petrelli et al, conducted a retrospective study using data from multiple databases to evaluate the relationship between HPV-16/18 and the incidence, risk, and prognosis of ESCC in adults. The study showed that approximately 1/5 of ESCC patients tested positive for HPV-16/18, with variations in prevalence among different regions (51). These reports and the analysis of differential genes suggested that ESCC with TLS and high expression of IgG4 may be associated with long-term chronic infections caused by viruses or microorganisms. Such infections may further promote tumor development and result in poor survival.

The most significant differentially expressed gene related to the cytokine-cytokine receptor interaction pathway is IL-17RE, which encodes the receptor protein for IL-17 cytokines (52). The IL-17 cytokine family has six members, initially thought to be mainly secreted by type 2 T helper cells (Th17 cells), but later found to be produced by other cell types such as γ, δ T cells, and NK cells. IL-17 plays various roles in the immune system, including promoting inflammation, mediating immune cell migration, and facilitating autoimmune reactions (52). There is currently no definitive report on the interaction between IgG4 and IL-17. Some research suggested a positive correlation between IgG4 and IL-17 (53, 54). One study showed that CXCL13 played an important role in the progression of hepatocellular carcinoma, significantly promoting the expression of IL-12 and IL-17 and inducing B cell secretion of IgG4 (54). In a study of Crohn’s disease, IL-17 and IL-21 produced by Th17 cells and T follicular helper cells (Tfh cells) were found to induce the differentiation of IgG4 containing plasma cells (53). However, other studies suggested a negative correlation between IgG4 and IL-17, where an increase in IgG4 levels inhibited IL-17 expression (55). In a study on IgG4-related dacryoadenitis and sialadenitis (IgG4-DS), IL-17 was rarely detected in biopsy tissues from patients with IgG4-DS (55). In this study, the group with high IgG4 expression and low IgG1 expression had more infiltrations of activated NK T cells, activated DC cells, and plasmacytoid DC cells compared to the group with high IgG1 expression and low IgG4 expression, while there was no statistically significant difference in Th17 cells. Based on our results and previous reports, it can be speculated that the higher expression of IL-17 in ESCC with TLS and high IgG4 expression might be associated with an increased presence of NK cells and NK T cells. However, the specific biological functions of IgG4 and IL-17, as well as the mechanisms underlying their interaction, require further investigation.

In summary, this study elucidates the cellular composition and morphological characteristics of TLS in ESCC, the expression of TLS-related Ig subtypes, and the association between the distribution of TLS and clinical prognosis. It indicates that ESCC with TLS may have better survival than those without, and ESCC with TLS and high IgG4 expression may lead to different immune cell infiltration patterns and poor prognosis. Analysis of TLS-related gene expression profiles in ESCC suggests that high expression of TLS-related IgG4 may be associated with long-term chronic infection or certain cytokines productions, such as IL-17, further affecting the occurrence, development, and treatment efficacy of tumors. These results contribute to a deeper understanding of the relationship between TLS and TIME in ESCC, identifying potential targets for tumor immunotherapy and providing basic data and theoretical basis for further optimizing immunotherapy strategy of ESCC. TLS are emerging as promising targets for immunotherapy. Thus, understanding the complexity and heterogeneity of TLS may help elucidate individual differences in immunotherapeutic response and facilitate the development of more precise therapeutic regimens.

## Data Availability

The raw data supporting the conclusions of this article will be made available by the authors, without undue reservation.
