# Supplementary material for: Increased IgG4 expression within tertiary lymphoid structures of esophageal cancer and implications for prognosis
Source: Front Immunol. 2025 Sep 18;16:1654655. doi: 10.3389/fimmu.2025.1654655 (PMC12488685; doi:10.3389/fimmu.2025.1654655)
Supplement: Supplementary file 1 [file DataSheet1.docx]

Supplementary materials

# Clinical cohorts and specimens

The criteria for inclusion of ESCC cases include: (1) Patients who have undergone esophageal radical surgery or subtotal resection surgery and have been pathologically diagnosed as ESCC; (2) Absence of any history of preoperative chemotherapy or radiotherapy; (3) No concurrent malignant tumors or immune system disorders; (4) Availability of fundamental clinical data and survival prognosis information; (5) Availability of FFPE tissue or slides. The collected basic clinical information of enrolled patients includes: name, gender, age, histological type, and TNM staging. The TNM staging is determined according to the 8th edition of the American Joint Committee on Cancer (AJCC) staging system, where T staging is determined by the depth of invasion of the primary esophageal tumor, N staging is based on the number of regional lymph node metastases, and M staging is assessed with the presence or absence of distant organ metastasis. Additionally, the degree of differentiation is classified as G1 (well-differentiated), G2 (moderately differentiated), or G3 (poorly differentiated/undifferentiated). The pathological progression type is categorized as medullary, protruding, mushroom, ulcerative, intracavitary/cauliflower, or constrictive type. The study also examined other various characteristics of tumors, including their location (cervical segment, upper thoracic segment, middle thoracic segment, lower thoracic segment, multiple locations), depth of invasion (encompassing the mucosal sublayer, submucosal layer, superficial muscular layer, deep muscular layer, and outer layer), and dimensions (length, width, height, and volume). Additionally, patient histories of smoking, alcohol consumption, and family medical background were considered.

# Statistical methods

For comparison of the distributions between two independent samples that follow a normal distribution, the Levene's test for equality of variances was used prior to conducting a t-test. For comparison of the distributions between two independent samples that did not follow a normal distribution, the non-parametric Mann-Whitney U test was used instead of the t-test. For comparison of the distributions between multiple independent samples that do not follow a normal distribution, the Kruskal-Wallis test was used as a non-parametric alternative to ANOVA. For pairwise comparisons between multiple independent samples that did not follow a normal distribution, Dunn's multiple comparison test was used. For testing the independence hypotheses between two categorical variables, the non-parametric chi-square test was used. The Pearson chi-square test was used when the expected frequencies were greater than 5, while Fisher's exact chi-square test and Monte Carlo simulation method with 100,000 simulations were used when the expected frequencies were less than 5. For the correlation analysis between multiple independent samples that did not follow a normal distribution, Spearman's correlation analysis was used. The relationship among clinical indicators, the presence of TLS, TLS score, GC score, IgG4 group, and survival prognosis was analyzed using the single-factor COX regression analysis method. The Wald test and log-rank test were used to calculate the Hazard Ratio (HR) and Concordance Index (C-Index) between different groups of categorical variables. Kaplan-Meier survival curves were plotted for statistically significant indicators and further analyzed using a multivariate COX proportional hazards model.

# The antibody information involved in this study.

Table S1. Antibody details


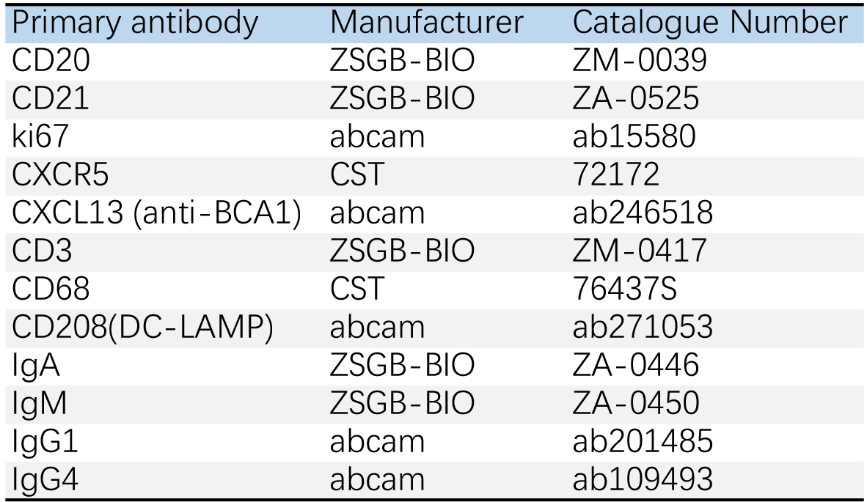


# The characteristics of lymphoid structures in ESCC

In the context of ESCC, tumor progression is often associated with lymph node enlargement or metastasis. We conducted staining analyses of critical structures within the lymph nodes of esophageal cancer tissues, as illustrated in Figure S1.


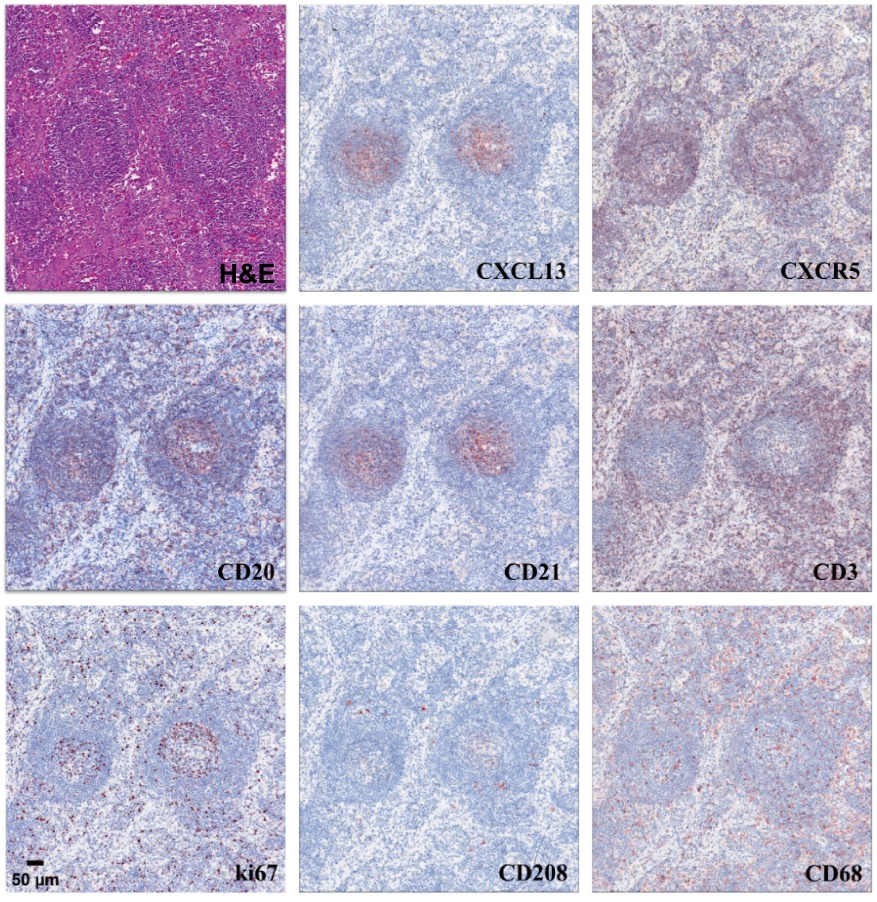


Fig.S1 The composition of lymphoid follicles in ESCC lymph nodes

# Kaplan-Meier survival analysis on some important clinical indicators

Furthermore, Kaplan-Meier survival analyses were performed on several key clinical indicators, including T staging, N staging, history of alcohol use, pTNM staging, depth of infiltration, and the presence of TLS. These traditional clinical indicators are crucial for prognostic evaluation (Figure S2 A-G). Our findings also confirmed that the presence of TLS is indicative of a more favorable prognosis, as evidenced by overall survival (OS) and progression-free survival (PFS) statistics (Figure S2 H, I).


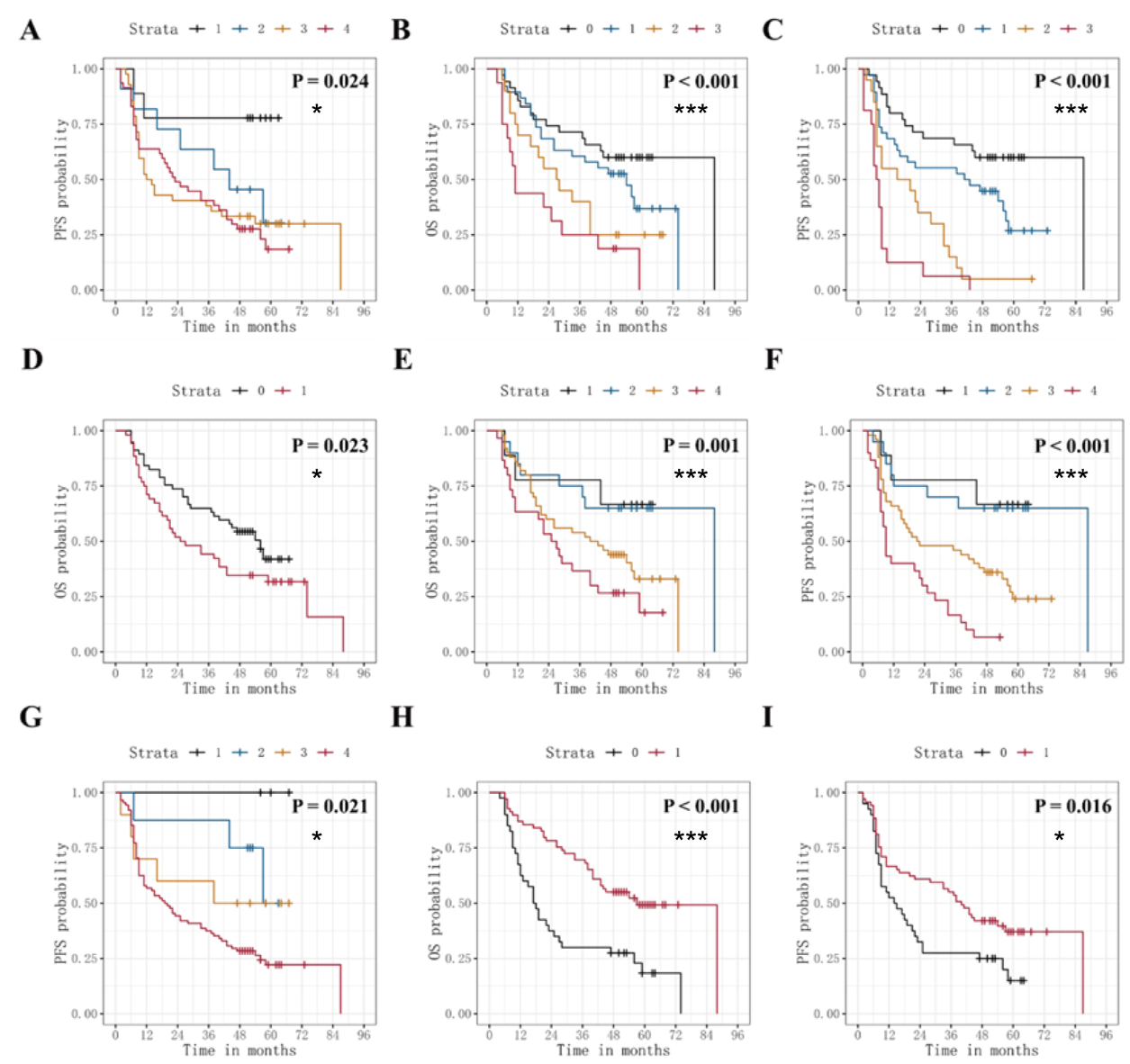


Fig.S2 Kaplan-Meier survival curves for various clinical indicators of ESCC (A: T staging; B, C: N staging; D: Alcohol history, where 0 represents no alcohol history and 1 represents a history of alcohol consumption; E, F: pTNM staging; G: Depth of infiltration, where 1 represents the mucosal layer, 2 represents the submucosal layer, 3 represents the muscularis propria, and 4 represents the adventitia; H, I: TLS (Tertiary Lymphoid Structures), where 0 indicates the absence of TLS and 1 indicates the presence of TLS; The p-value represents the results of the log-rank test.)

1. Kaplan-Meier survival curves of TLS score and GC score.


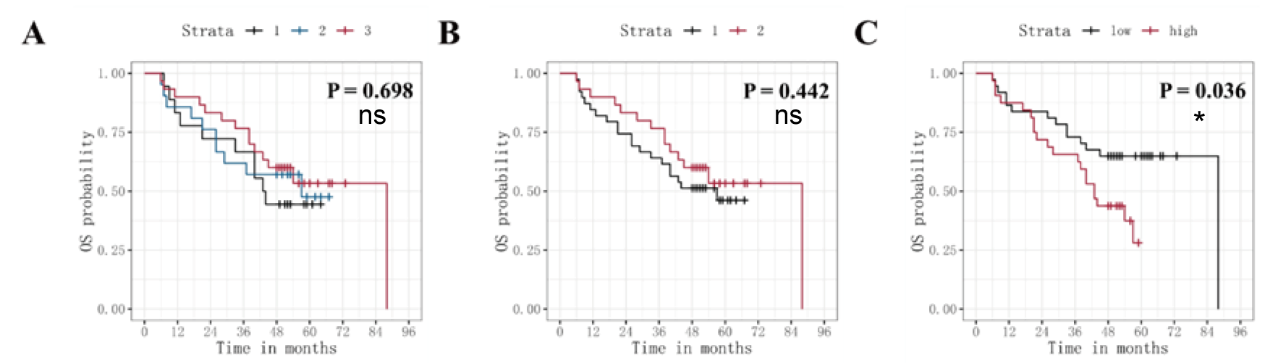


Fig.S3 Kaplan-Meier survival curves for various score indicators of ESCC (A: TLS score; B: GC score; The p-value represents the results of the log-rank test)

# Univariate and multivariate Cox regression analysis.
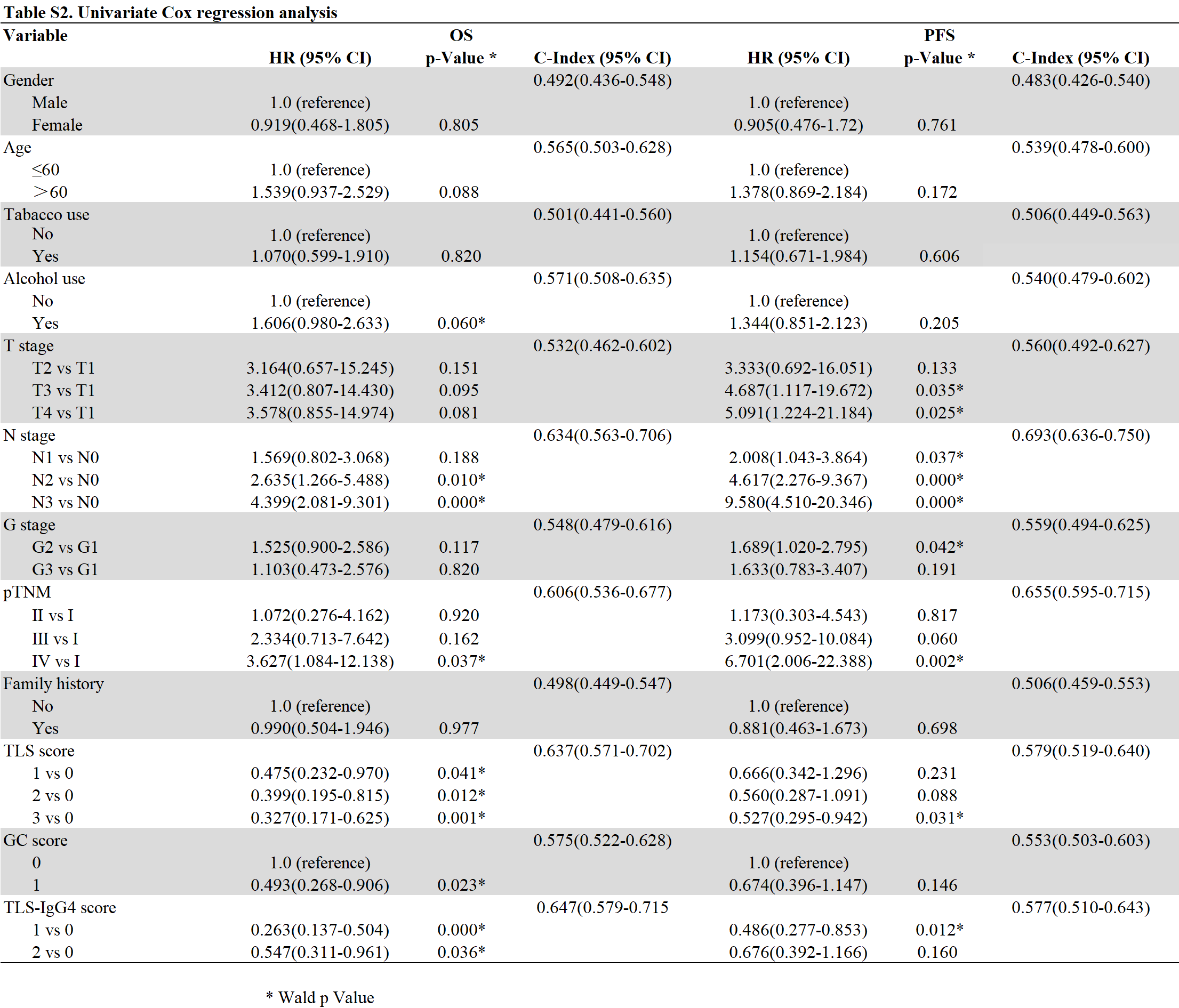


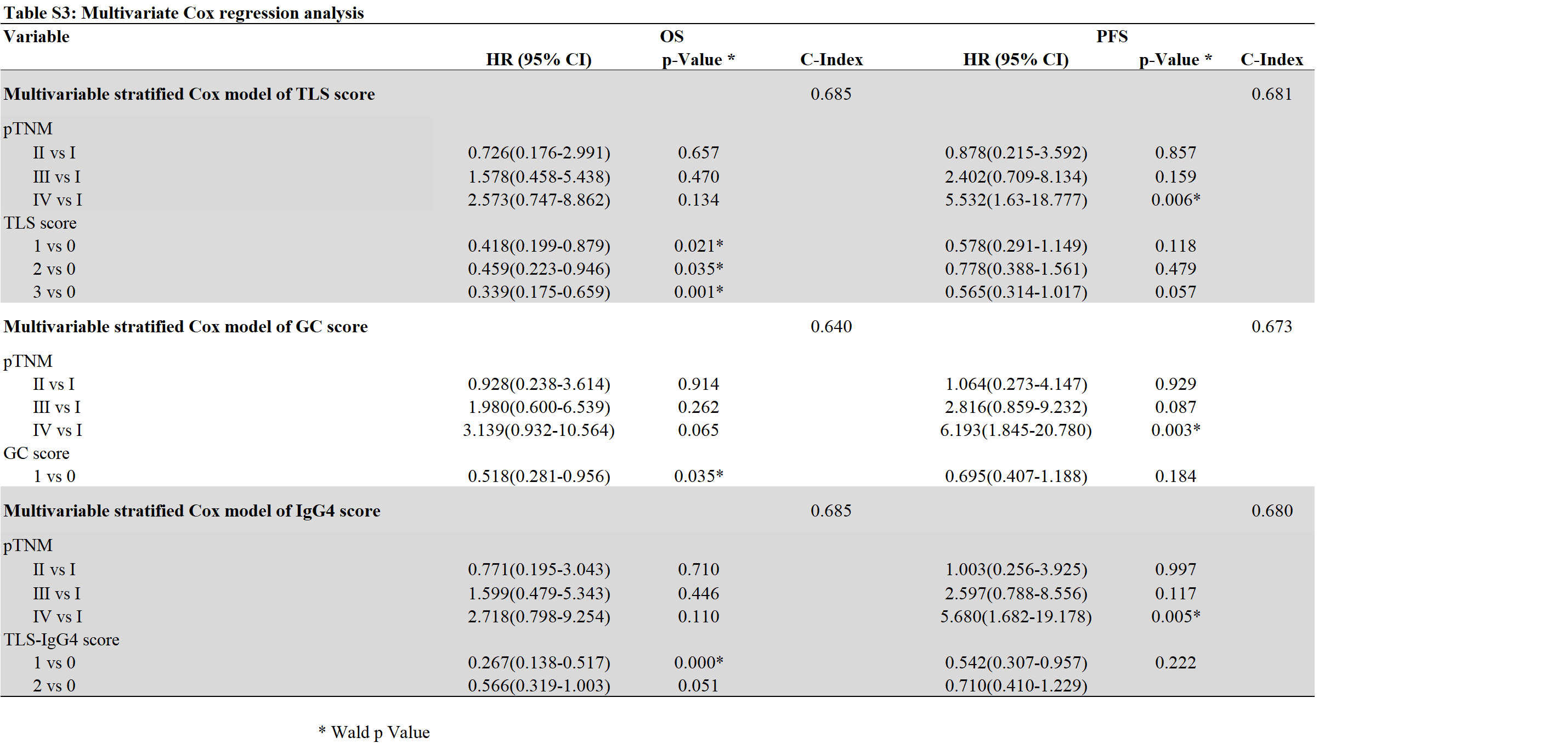


# The gene expression profile of TLS in ESCC GEO database cohort


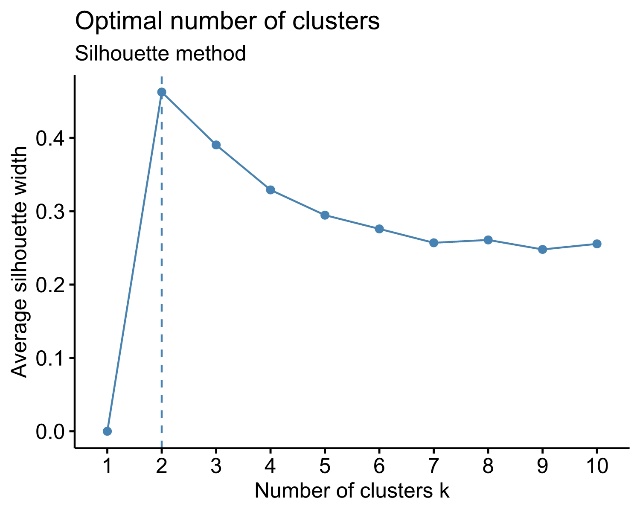


Fig.S4 Clustering diagram using the silhouette coefficient method


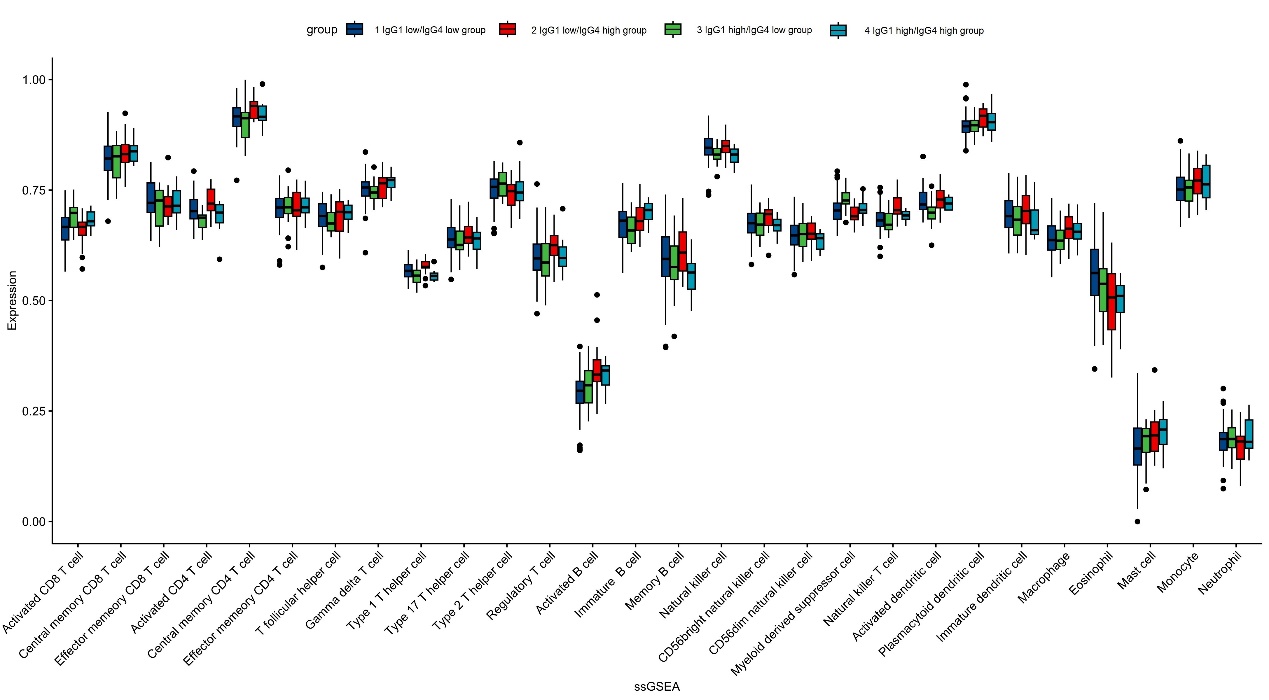


Fig.S5 A comparative analysis of the differential expression of 28 immune cells among different Ig subgroups


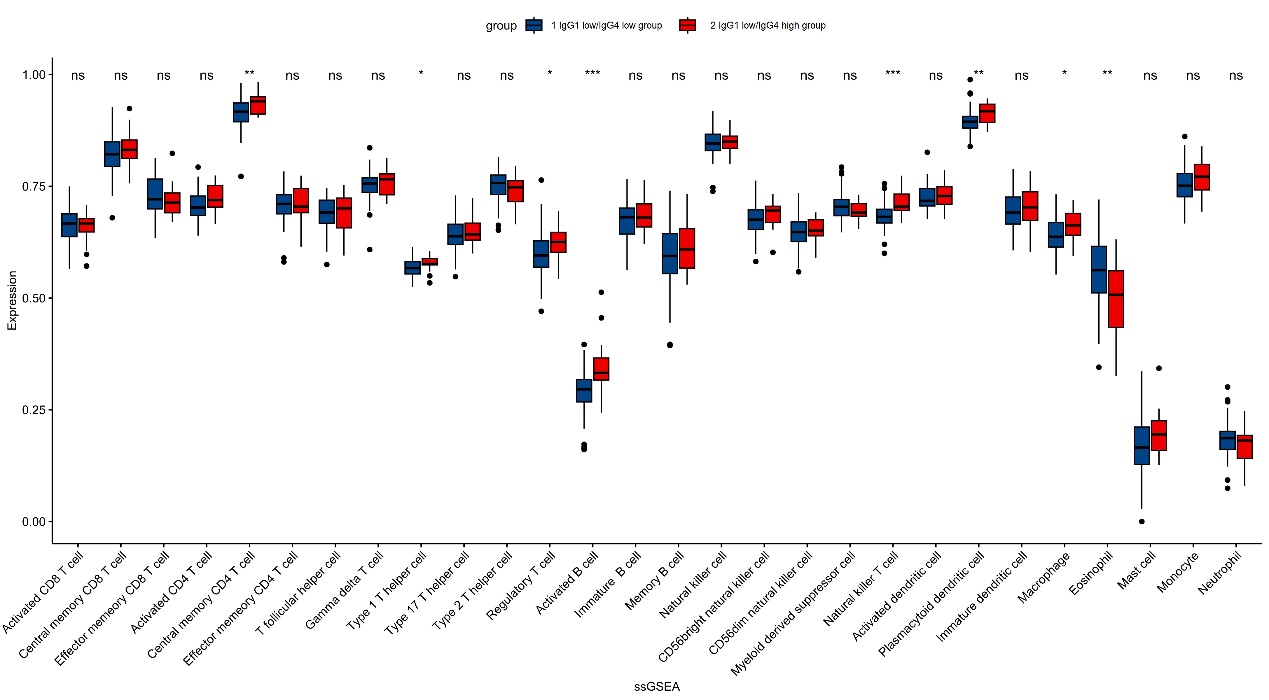


Fig.S6 The differential expression of 28 immune cells between the “1 IgG1 low/IgG4 low group” and “2 IgG1 low/IgG4 high group”


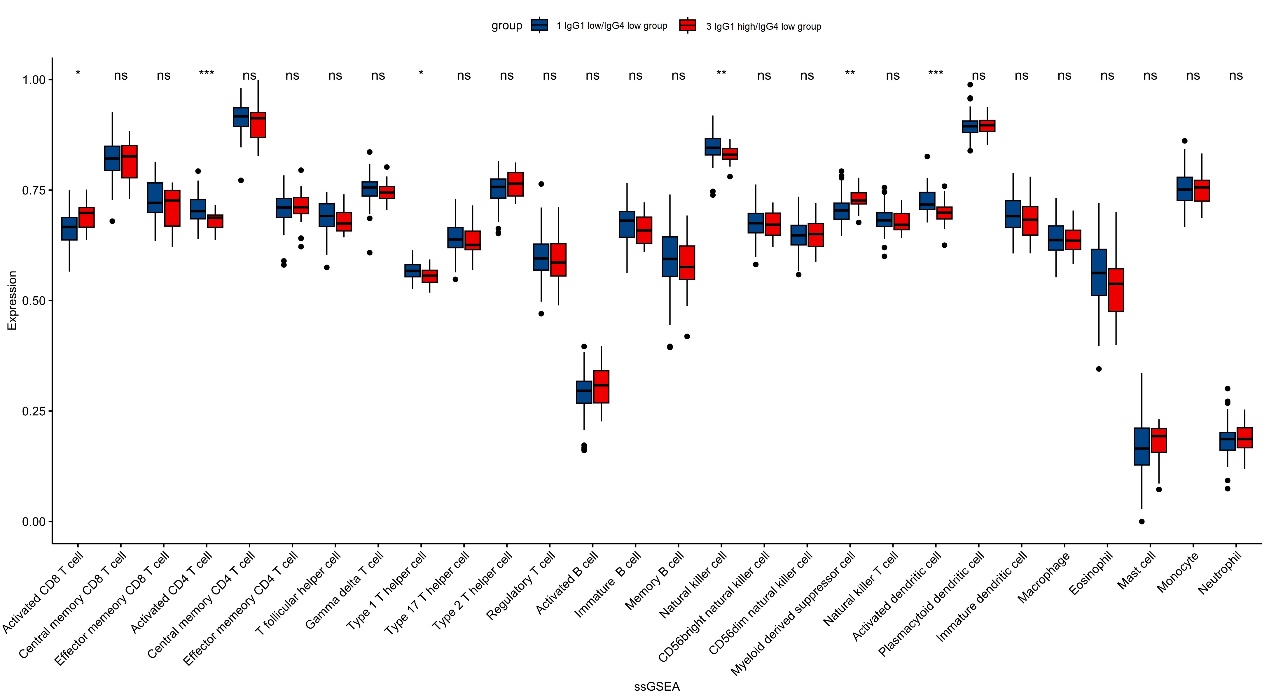


Fig.S7 The differential expression of 28 immune cells between the “1 IgG1 low/IgG4 low group” and “3 IgG1 high/IgG4 low group”


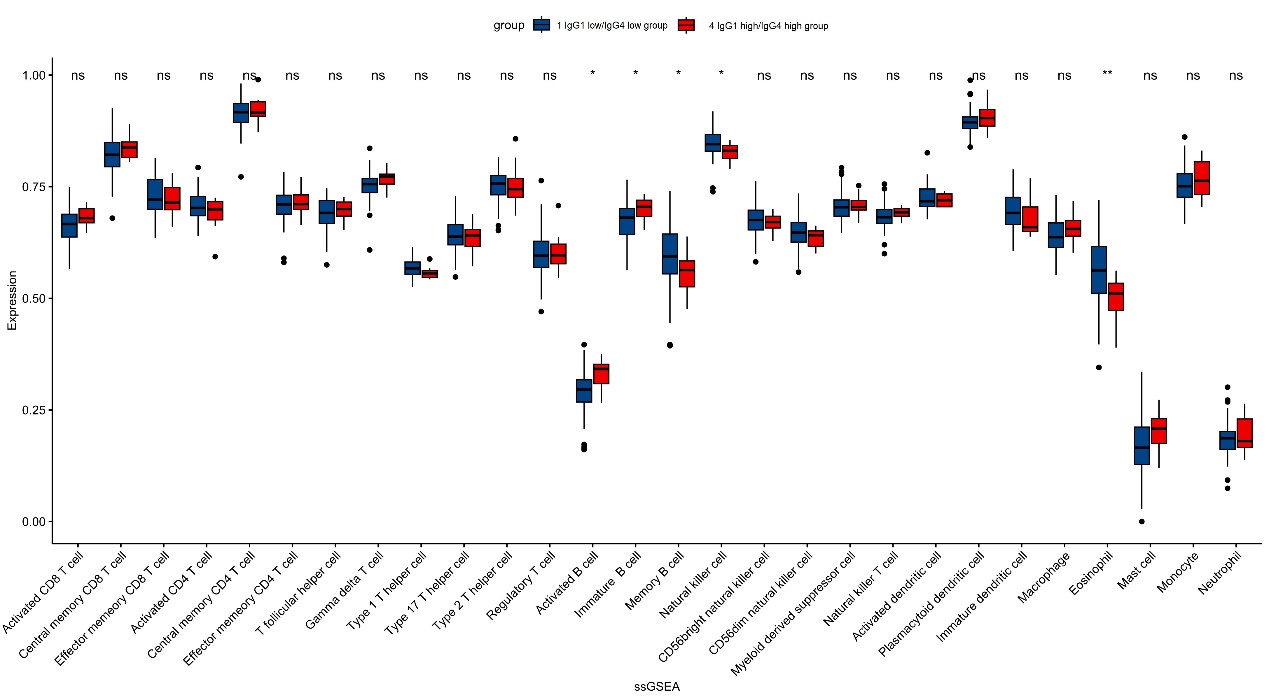


Fig.S8 The differential expression of 28 immune cells between the “1 IgG1 low/IgG4 low group” and “4 IgG1 high/IgG4 high group”


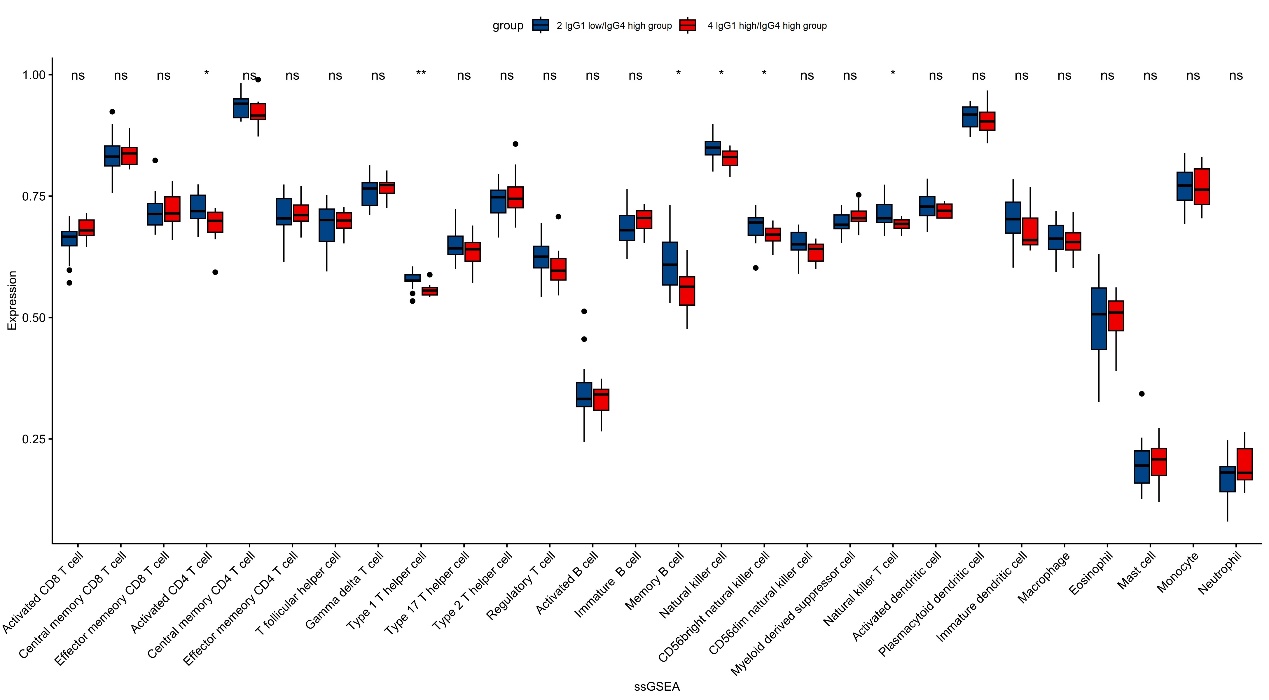


Fig.S9 The differential expression of 28 immune cells between the “2 IgG1 low/IgG4 high group” and “4 IgG1 high/IgG4 high group”


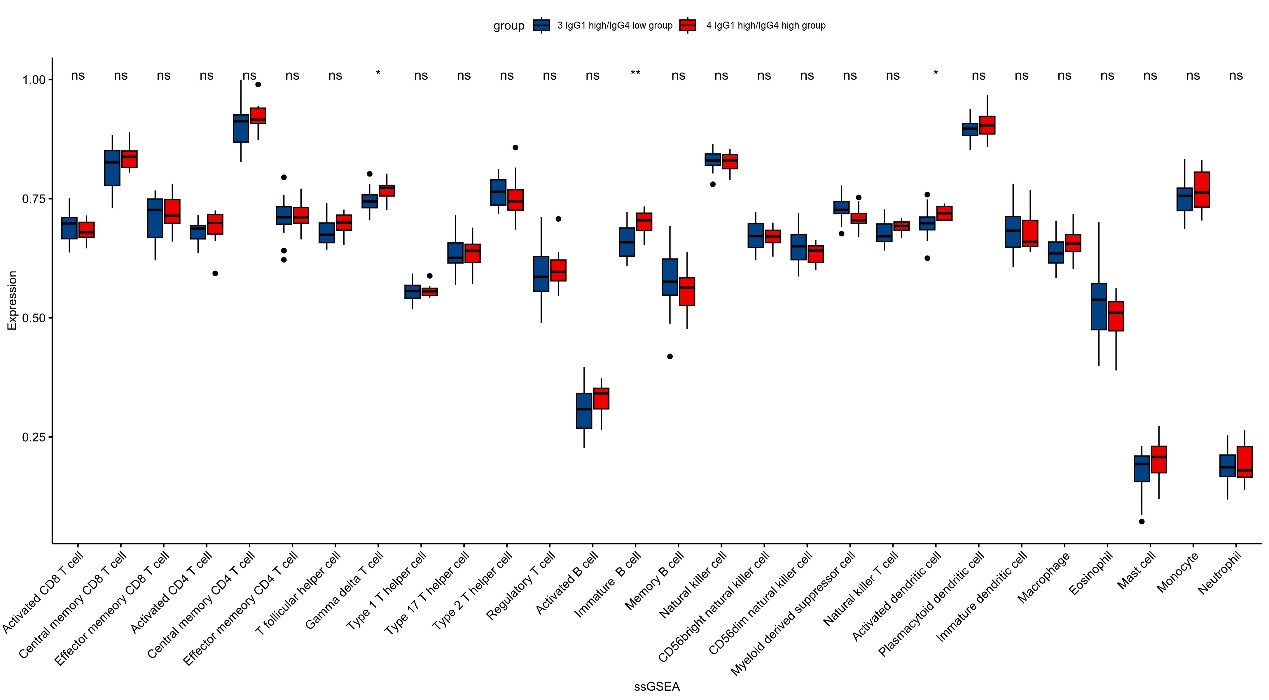


Fig.S10 The differential expression of 28 immune cells between the “3 IgG1 high/IgG4 low group” and “4 IgG1 high/IgG4 high group”
